# HIV-Associated Neurocognitive Impairment in the Modern ART Era: Are We Close to Discovering Reliable Biomarkers in the Setting of Virological Suppression?

**DOI:** 10.3389/fnagi.2019.00187

**Published:** 2019-08-02

**Authors:** Alessandra Bandera, Lucia Taramasso, Giorgio Bozzi, Antonio Muscatello, Jake A. Robinson, Tricia H. Burdo, Andrea Gori

**Affiliations:** ^1^Infectious Disease Unit, Department of Internal Medicine, Fondazione IRCCS Ca’ Granda Ospedale Maggiore Policlinico, Milan, Italy; ^2^Department of Pathophysiology and Transplantation, University of Milano, Milan, Italy; ^3^Infectious Diseases Clinic, Department of Health Sciences, School of Medical and Pharmaceutical Sciences, Policlinico Hospital San Martino, University of Genova (DISSAL), Genova, Italy; ^4^Department of Neuroscience, Lewis Katz School of Medicine at Temple University, Philadelphia, PA, United States

**Keywords:** marker, neurocognitive impairment, HIV, Art, ANI, HAND, AIDS, dementia

## Abstract

The prevalence of the most severe forms of HIV-associated neurocognitive disorders (HAND) is decreasing due to worldwide availability and high efficacy of antiretroviral treatment (ART). However, several grades of HIV-related cognitive impairment persist with effective ART and remain a clinical concern for people with HIV (PWH). The pathogenesis of these cognitive impairments has yet to be fully understood and probably multifactorial. In PWH with undetectable peripheral HIV-RNA, the presence of viral escapes in cerebrospinal fluid (CSF) might explain a proportion of cases, but not all. Many other mechanisms have been hypothesized to be involved in disease progression, in order to identify possible therapeutic targets. As potential indicators of disease staging and progression, numerous biomarkers have been used to characterize and implicate chronic inflammation in the pathogenesis of neuronal injuries, such as certain phenotypes of activated monocytes/macrophages, in the context of persistent immune activation. Despite none of them being disease-specific, the correlation of several CSF cellular biomarkers to HIV-induced neuronal damage has been investigated. Furthermore, recent studies have been evaluating specific microRNA (miRNA) profiles in the CSF of PWH with neurocognitive impairment (NCI). The aim of the present study is to review the body of evidence on different biomarkers use in research and clinical settings, focusing on PWH on ART with undetectable plasma HIV-RNA.

## Introduction

Thirty-seven million people are currently living with HIV infection in the world, according to World Health Organization estimates. A variable proportion of people with HIV (PWH), suggested up to 60%, might develop some form of HIV-associated neurocognitive disorder (HAND) in the course of their life (Schouten et al., 2011). HAND is categorized as three main conditions based on the severity of neurocognitive deficits: asymptomatic neurocognitive impairment (ANI), HIV-associated mild neurocognitive disorder (MND), and HIV-associated dementia (HAD; Antinori et al., 2007).

The neurocognitive impairment (NCI) underlying these conditions has a multifactorial etiopathogenesis, with many contributing factors. One of the most studied contributing factors is the uncontrolled replication of HIV in cerebrospinal fluid (CSF) and brain tissue, causing direct central nervous system (CNS) damage, further complicating the global picture of HAND. However, a certain grade of NCI has been found in PWH on stable antiretroviral treatment (ART) with persistently undetectable viremia, highlighting ongoing brain volume loss and white matter injury without association to HIV viral load (McMurtray et al., 2008; Cardenas et al., 2009; Gongvatana et al., 2009). These data suggest that the direct role of the virus alone cannot explain the presence of HAND and that other factors, such as chronic inflammation, cytokine release and cellular damage, could all be involved in the pathological process. Specifically, common autopsy findings in ART-treated PWH included brain endothelial cell activation and alterations in neurochemical synaptic transmission with a lymphocyte infiltrate rather than the monocyte dominant encephalitis observed in advanced pre-ART HIV disease.

Many factors have been studied so far to determine the risk of developing HIV-associated NCI, but, until now, a single marker has not been identified that effectively differentiates PWH with or without NCI. In PWH with uncontrolled HIV replication and HAND, therapy is optimized for controlling viral replication; however, there is no standardized consensus on therapy targeting HAND for PWH with optimal viral suppression. The identification of biomarkers with the potential for predicting or stratifying the risk for NCI could be pivotal for the early diagnosis of HAND and long-term management of chronically infected PWH, assessing the efficacy of therapeutic interventions along time. However, as ART-mediated viral suppression limits the role of HIV as a mechanism of CNS injury, potential biomarkers should be investigated between inflammation-linked or neuronal damage markers that could be influenced by different individual host characteristics, such as the genetic likelihood of Alzheimer’s, age, and comorbidities.

In this review article, we analyzed the current body of evidence concerning biomarkers of NCI in PWH on ART and with controlled HIV replication, investigating their applicability in the context of clinical practice. The aim of this review is to investigate which mechanisms are currently studied and possibly correlated to NCI, and whether some markers of neuronal damage or immune activation could be used for diagnostic purposes or even in the longitudinal follow-up of ART-treated PWH.

## Methods

This review article is based on the literature analyzing the markers of NCI in the course of ART-treated HIV infection. Our review examined all articles published in English and available on PubMed database until December 16, 2018. No restriction criteria based on year of publication were used. Online publications ahead of print were also included. The PubMed database was screened using the search terms: {(HIV) [AND NCI] AND marker} NOT review [Publication Type]. One-hundred and eleven articles published between August 1996 and December 2018 were initially selected and screened for eligibility based on title and abstract. With the aim of eliminating the confounding factor of uncontrolled CSF viral replication and viral escapes, we excluded studies performed in ART naïve subject. *In vitro* studies, post-mortem studies, and animal studies were also excluded. The remaining manuscripts were thereafter read in whole, and included in the review article, if they reported data on at least one possible marker of NCI, studied in PWH (at least partly) on ART.

**Figure 1:**
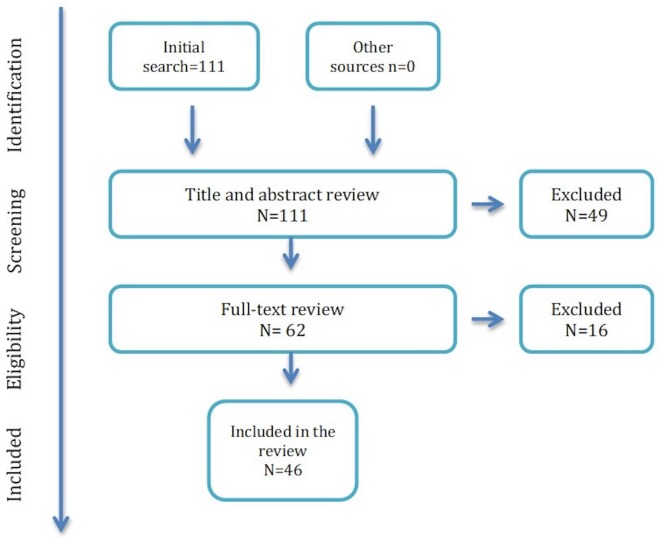
Flowchart for study selection.

Forty-three articles were excluded because they did not focus on any biomarker or only concerned viral markers; four because the studies were performed on animals; two were *in vitro* studies; four were post-mortem studies; eight were performed in ART-naïve PWH or PWH with CSF replicating HIV; four were reviews (Figure 1).

Forty-six articles were included in the final version of the review, encompassing 201 distinct hypotheses of correlation between different biomarkers and NCI.

References cited in the studies were also checked to identify other relevant articles not found in our initial search.

### Technical Limitations

One of the major issues linked to identification of reliable biomarkers of NCI is the reproducibility of the results. In fact, the techniques that are used to measure different biomarkers in different studies are often not fully comparable. ELISA-based assays, which are commonly used to detect and dose many biomarkers, have been originally developed to measure a single analyte and later developed as multiplex assays, allowing measurement of multiple analytes from a single sample. However, many different sources and kits from different manufacturers are now available and they might provide different results, while the same analyte can be measured differently in multiplex or single determinations. In addition, different kits can be designed for different ranges of values with different software and require different dilutions of the CSF or plasma, which can confound the data between studies. Also, the modern omics approach has similar limitations as well. The results of an omics study can vary with the manufacturer, the platform, and which version is used, owing to low overlap of results when the same samples are run on different platforms. In addition, the omics approach requires correction for multiple comparisons and discussion of fold change definitions, both of which are controversial within the fields.

Finally, statisticians often advocate log transformation of results that may be appropriate for some biomarkers but is problematic for others. Indeed, log transformation of results may obscure the marginal values of more labile analytes, whose values can be at the margin of detection, and leads to publication of findings driven by a very few positive values.

All those technical issues must be considered in the final considerations on the significance of the various analytes that have been studied in CNS.

## Markers of Neuronal Damage and Alteration of Signaling

Key events that follow invasion of CNS by HIV and contribute to HAND include direct neuronal apoptosis, dysregulation of neuronal support cells, and loss of synaptodendritic signaling. Identifying sensitive CSF biomarkers of HIV-induced neuronal damage seems essential for HIV/neuroAIDS staging, evaluation of response to treatment, and clarifying possible mechanisms of neural damage. We summarized data on available neuronal damage and alteration of signaling biomarkers, underlying the main strengths and limitations of the available literature on the topic (Table 1).

**Table 1:** Biomarkers of neuronal damage and correlation with neurocognitive impairment (NCI) in people living with HIV.

Biomarker	Body fluid	Findings	Research gaps	Reference
β**-Amyloid 42**				
	CSF	No correlation with white matter hyperintensity in ART-treated PWH.	Lack of randomized trials; Lack of longitudinal data; little data in course of ART	Trentalange et al. (2018)
	CSF	No correlation with abnormalities in the periventricular white matter.		Steinbrink et al. (2013)
	CSF	Significant correlation with neuropsychological tests score:		Steinbrink et al. (2013)
		-HIV Memorial Sloan–Kettering scale score		
		-Not with HIV dementia scale (HDS) and the Mosaic test (MT) scores		
	CSF	NO correlation with GDS.		Cysique et al. (2015)
ε4 APOE carriage		Association with lower CSF Aβ1-42 concentrations. NO correlation with NCI.	Association with NCI not proven in HIV	Bertram et al. (2008)
ε4 APOE carriage		No association with HAND in HIV.		Bora et al. (2014) and Cysique et al. (2015)
**Neurofilament proteins**
	Plasma	Higher concentration in worse cognitive performance.	No data in correlation with experimental neuroprotective therapies. Lack of randomized trials.	Anderson et al. (2018)
	CSF	Independent biomarker of baseline NCI status.		Guha et al. (2019)
	CSF	Negative association with cognitive performance.		Jessen Krut et al. (2014) and Anderson et al. (2018)
	CSF	Trend towards higher levels in aviremic PWH with NCI compared to others.		Edén et al. (2016)
	CSF	Higher levels in NCI vs. unimpaired PWH.		Guha et al. (2019)
	CSF	High levels in untreated neuroasymptomatic PWH.		Jessen Krut et al. (2014)
	CSF	Predictive of HAD development in neuroasymptomatic PWH.		Gisslén et al. (2007)
	CSF	Low levels in ART-treated PWH and in PWH.		Jessen Krut et al. (2014)
	CSF	Levels decline after starting ART.		Jessen Krut et al. (2014)
	CSF	Levels remain stable in course of stable ART.		Sacktor et al. (2018)
	CSF	Similar levels in course of ART in early treated PWH compared to others.		Oliveira et al. (2017)
	CSF	Correlation with neopterin levels.		Edén et al. (2016)
	CSF	Direct correlation with C1q in CSF.		McGuire et al. (2016)
**Calcium binding protein B (S100B)**
	CSF	Higher levels in NCI compared to unimpaired subjects.	Little data, lack of longitudinal studies. Lack of randomized trials.	Yuan et al. (2017) and Guha et al. (2019)
**Tau protein**
	CSF	Higher mean values in PWH with moderate or severe abnormalities of periventricular white matter.	Little longitudinal data, absence of a defined cut-off for defining NCI, little use in the follow-up of PWH on ART. Lack of randomized trials.	Steinbrink et al. (2013)
	CSF	No correlation with white matter hyperintensities in ART, weak correlation off ART.		Trentalange et al. (2018)
	CSF	Significant correlation with neuropsychological test score:		Steinbrink et al. (2013)
		-Memorial Sloan–Kettering scale score		
		-HIV dementia scale score		
		-Mosaic test score		
	CSF	Normalization after improvement of neurocognitive function in a single case report.		Andersson et al. (2006)
	CSF	No changes in virologically suppressed PWH after switch to ART regimens with enhanced central nervous system penetrability.		Tiraboschi et al. (2015)
Phospho-tau	CSF	Significant correlation with GDS.		Cysique et al. (2015)
**LIPIDS**				
Sphingomyelin:ceramide ratio	CSF	Greater sphingomyelin:ceramide ratios for acyl chain lengths of C16:0, C18:0, C22:0, and C24:0 were associated with worse performance on several indices of memory.	Lack of randomized trials; Lack of longitudinal data	Mielke et al. (2010)
Sphingomyelin:cholesterol ratios	CSF	Higher sphingomyelin:cholesterol ratios were significantly associated with worse performance on the Rey Auditory Verbal Learning Test trail 5 score (*p* = 0.018) and delayed recall.		Mielke et al. (2010)
**Extracellular vesicles**				
	CSF	Higher in HIV + that in uninfected subjects.	Little studied, still no longitudinal data, absence of solid correlations in ART-treated PWH. Lack of randomized trials	Guha et al. (2019)
	CSF	Higher levels in NCI compared to unimpaired subjects.		Guha et al. (2019)
	CSF	Direct correlation with S100B.		Guha et al. (2019)
**Wnt pathway**				
DKK1	Plasma	Higher levels in NCI compared to unimpaired subjects, particularly in PWH taking ART with HIV RNA levels ≤50 copies/mL.	Little studied, still no longitudinal data, absence of solid correlations in ART-treated PWH. Lack of randomized trials.	Yu et al. (2017)
**Mitochondrial DNA**				
	CSF	Higher levels in NCI compared to unimpaired subjects.	Contradictory data, lack of reliable data in ART-treated PWH; lack of longitudinal data. Lack of randomized trials.	Pérez-Santiago et al. (2016)
	CSF	Higher levels in better neurocognitive performance.		Pérez-Santiago et al. (2017)
	CSF	Inverse correlation with NFL levels.		Pérez-Santiago et al. (2017)
	CSF	Association with biomarkers of inflammation (IP-10 in CSF and MCP-1 in plasma).		Pérez-Santiago et al. (2016)
	CSF	Higher levels in individuals with detectable CSF HIV RNA and in the absence of ART.		Mehta et al. (2017)
	CSF	Higher levels in PWH with mild NCI than in asymptomatic subjects.		Mehta et al. (2017)
	CSF	Direct association with IL-6; inverse association with ceruloplasmin, transferrin, and VEGF.		Mehta et al. (2017)
	CSF	Direct association with MCP-1 in CSF, TNF-α and IL-8 in plasma.		Pérez-Santiago et al. (2017)
Different mitochondrial haplogroup carriage		No correlation with IL-6, IL-8, IP-10, and TNF-α in CSF in African and Hispanic ancestry. Haplogroup H had significantly lower CSF TNF-α levels in European ancestry with suppressed plasmatic HIV-RNA viremia.		Samuels et al. (2016)

*Abbreviations: ART, antiretroviral treatment; GDS, global deficit score, CSF, cerebrospinal fluid; PWH, people with HIV*.

### β-Amyloid 42

β-Amyloid 42 (Aβ42) is the major component of brain amyloid plaques in the course of Alzheimer’s disease, and may activate mast cells, which could play an important role in Alzheimer’s disease pathogenesis (Niederhoffer et al., 2009). Patients without clinical cognitive impairment with Aβ42 pathology are considered at high risk of preclinical Alzheimer’s disease (Sperling et al., 2011), cognitive decline (Petersen et al., 2016), and progression to mild cognitive impairment (Knopman et al., 2012). Aβ42 levels in CNS have been previously linked to HIV-RNA load, indicating that HIV replication in the brain could be an active driver of neuroinflammation and of abnormal protein clearance (Levine et al., 2016). However, few studies have focused on Aβ42 levels with successful suppression of HIV replication. CSF levels of Aβ42 from ART-naïve PWH have been previously correlated with average white matter hyperintensities (ρ = 0.319, *p* = 0.045; Trentalange et al., 2018) that, in turn, have been associated with cognitive impairment (Mok and Kim, 2015). However, such correlation was not confirmed in ART-treated PWH (Trentalange et al., 2018), and a study conducted in 94 PWH (72% with HIV-RNA undetectable) did not find a correlation between CSF Aβ42 levels and abnormalities in the periventricular white matter (Steinbrink et al., 2013). In the same study, the CSF Aβ42 was correlated to scores of different neuropsychological tests, and Aβ42 correlated significantly with HIV Memorial Sloan–Kettering scale (MSKS) score but not with HIV dementia scale (HDS) and the Mosaic test (MT) scores (Steinbrink et al., 2013). Moreover, in ART-treated PWH with undetectable HIV RNA in their plasma or CSF, CSF Aβ42 levels did not correlate with clinically relevant levels of impairment, defined in accordance with the Global Deficit Score (GDS) method, or with past diagnosis of HAND (Cysique et al., 2015). Also, the ε4 allele of Apolipoprotein E (APOE), an apolipoprotein thought to be partially responsible for amyloid clearance in the CNS and a risk factor for late-onset sporadic Alzheimer’s disease (Bertram et al., 2008), showed no association with HAND in HIV (Cysique et al., 2015).

Although Aβ42 could probably be used in other settings, including cognitive impairment in HIV viremic PWH, current evidences on its use in controlled HIV infection are still limited and do not offer additional information on cognitive evaluation and staging in the clinical practice.

### Neurofilament Proteins

The light subunit of the neurofilament protein (NFL) is a major structural element of myelinated axons that is crucial for maintaining the axonal caliber and facilitating an effective nerve conduction (Hoffman et al., 1987). Elevated NFL levels are found in CSF of PWH when this structural component is released during neuronal and axonal injury, which has been reported in several neurological disorders including HAND (Abdulle et al., 2007; Jessen Krut et al., 2014; Peterson et al., 2014; McGuire et al., 2015; Gisslén et al., 2016; Yilmaz et al., 2017). In HIV-infected adults, higher plasma NFL concentration has been significantly associated with worse neuropsychological performance, after adjustment for possible confounding factors including age, CD4+ T cell count, and plasma HIV RNA levels (Anderson et al., 2018). High NFL concentration in CSF was found to be an independent biomarker of baseline NCI status in multivariable linear regression models adjusted for age, race, and plasma viral load (Guha et al., 2019). CSF NFL levels were also found to correlate with C1q concentrations in CSF in ART naïve PWH. The complement system (C1q/C3) is a key mediator of synaptic pruning during normal development and HIV inappropriately induces C1q and C3 production in the brain. In a 2016 study of 40 PWH with 58% of PWH on ART, a nearly significant elevation of CSF C1q expression was observed in cognitively impaired subjects between 18 and 24 years old compared to cognitively normal subjects, and within the PWH group, CSF C1q correlated with CSF NFL levels (McGuire et al., 2016). However, the association between GDSs and CSF NFL has been observed mostly in individuals not receiving ART, in which plasma NFLs are higher compared to PWH receiving ART and usually decline over time after ART initiation (Andersson et al., 2006; Jessen Krut et al., 2014; Anderson et al., 2018). A trend towards higher CSF NFL was also seen in aviremic PWH with NCI compared to others (Edén et al., 2016) and, in a work including mostly ART-treated PWH (67 PWH of which 67% aviremic), CSF NFL levels were higher in PWH with NCI compared to unimpaired HIV-infected subjects (*p* < 0.05; Guha et al., 2019). In the same study, CSF NFL was higher in NCI (*p* = 0.009) and HAND (*p* = 0.007) but not in unimpaired HIV-infected subjects compared to HIV-uninfected controls (Guha et al., 2019). In another interesting study, high levels of CSF NFL were consistently found in HAD subjects and in untreated asymptomatic PWH, especially those with lower counts of CD4+ T lymphocytes, suggesting a subclinical axonal injury also in neuroasymptomatic PWH and not on treatment (Jessen Krut et al., 2014). Importantly, higher CSF NFL levels were found in neuroasymptomatic PWH compared to CD4-matched controls in a previous study, in which enrolled PWH were not on ART or, in some cases, treated with old drug combinations, which would now be considered suboptimal, and had with elevated median CSF HIV-RNA levels in both cases and controls (Gisslén et al., 2007). In a cross-sectional comparison between ART-treated PWH and HIV-uninfected subjects, only a minority of subjects [7/85 (8%) and 4/204 (2%), respectively] had high CSF NFL levels (Jessen Krut et al., 2014), and similar NFL concentrations were found in PWH under combined ART and in HIV-negative subjects 3.9 years older (Jessen Krut et al., 2014). While ART usually reduces CSF NFL concentrations in naïve subjects (Jessen Krut et al., 2014) in PWH with NCI and on stable ART, NFL does not seem to change significantly after 24 weeks (Sacktor et al., 2018). Moreover, ART-treated HIV aviremic subjects had similar CSF NFL concentrations either if treated early or late (after median 1.8 vs. 17.2 months) for HIV infection (Oliveira et al., 2017). In conclusion, NFL is a sensitive biomarker for axonal injury in several neurological disorders including HAND (Abdulle et al., 2007; Gisslén et al., 2007, 2016; Yilmaz et al., 2017), and the correlation found between CSF NFL and neopterin in NCI subjects suggests an association between NCI, CNS inflammation, and neuronal damage (Edén et al., 2016). NFL is one of the markers with a more consistent correlation with NCI in HIV; however, a longitudinal evaluation of NFL in ART-treated aviremic PWH seems not to be informative.

### Calcium Binding Protein B

Calcium binding protein B (S100B) is secreted by astrocytes and oligodendrocytes and can enter the extracellular space or bloodstream after spilling from injured cells. For this reason, S100B is considered a peripheral marker of CNS damage and blood-brain barrier permeability (Blyth et al., 2009; Kazmierski et al., 2012; Wu et al., 2016). Glial responses in HIV-infected individuals can be detected by elevation of CSF S100B (Green et al., 1999; Pemberton and Brew, 2001; Du Pasquier et al., 2013; Abassi et al., 2017) and its levels have been found to be higher in the CSF of PWH with NCI compared to unimpaired HIV-infected subjects (*p* < 0.05; Guha et al., 2019). In particular, CSF S100B was higher in NCI (*p* = 0.023) and HAND (*p* = 0.002) but not in unimpaired HIV-infected subjects compared to uninfected controls in a recent study conducted by Guha et al. (2019). However, in that study, SB100 was not confirmed as an independent biomarker of baseline NCI status in multivariable linear regression models adjusted for age, race, and plasma viral load (Guha et al., 2019). In another study, significantly higher levels of S100B were found in CSF of PWH with NCI compared with PWH without impairment (*p* = 0.0023); however, the analysis was unadjusted for possible confounding factors (Yuan et al., 2017). Definitive studies confirming the role of SB100 in the evaluation of NCI in the course of aviremic HIV infection are still lacking.

### Tau Protein

Total-tau and phospho-tau are considered reliable markers of clinically relevant neurodegeneration. Tau is a protein implicated in the assembly and stability of microtubules, while phospho-tau, its hyper-phosphorylated form, is produced in the course of inflammation and detaches from microtubules, forming neurofibrillary tangles (Calcagno et al., 2016). In a previous study performed in PWH (72% with HIV-RNA <200 copies/ml), higher mean values of total tau have been found in PWH with moderate or severe abnormalities of periventricular white matter than in PWH with no or mild abnormalities (*p* < 0.001 and *p* = 0.006, respectively; Steinbrink et al., 2013). The same findings were also confirmed in PWH with white matter abnormalities in the area of the basal ganglia, who showed significantly higher total tau levels (*p* = 0.0013) compared to PWH with no, or mild, abnormalities (*p* = 0.027; Steinbrink et al., 2013). On the other side, in ART-treated PWH, tau CSF levels did not correlate with the presence of hyperintensities in the white matter, which has been in turn associated with NCI, while a weak correlation was seen in naive PWH (Trentalange et al., 2018). The correlation among total tau level in CSF and scores of different neuropsychological tests has also been evaluated. Total tau correlated with the MSKS score (*r* = 0.252; *p* = 0.018), HDS score (*r* = 0.268; *p* = 0.015), and the MT score (*r* = 0.229; *p* = 0.036). Interestingly, none of those scores correlated with phospho-tau levels (Steinbrink et al., 2013). On the other hand, greater levels of NCI, evaluated by the GDS, were associated with higher CSF levels of phospho-tau (*r* = 0.10; *p* = 0.03), but NCI showed only borderline association at the univariate analysis (*p* = 0.05) to CSF total-tau levels, not confirmed at multivariable analysis (Cysique et al., 2015).

Although the normalization of CSF-tau has been correlated to improvement of neurocognitive function in a single case of a person initiating ART (Andersson et al., 2006), no changes in tau levels were found in studies of switch therapy in virologically suppressed PWH switching to ART regimens with enhanced CNS penetrability (Tiraboschi et al., 2015). In summary, tau CNS levels showed good correlation with both neuroimaging features and neuropsychological tests evocative of NCI in different studies. Data are still lacking on the possibility of use of this marker in the follow up of NCI.

### Lipid Biomarkers

During the course of CNS disorders, a disruption in the lipid metabolism may occur in the CNS, resulting in elevated CSF levels or brain accumulation of lipid biomarkers. Indeed, levels of ceramide and sphingomyelin are significantly increased in brain tissues and CSF of PWH with dementia (Haughey et al., 2004). This could be due to an induction of the lipid metabolism caused by the cytokines produced by glial cells. Moreover, an overproduction of ceramide, resulting from oxidative assault on lipid membranes in the CNS, can be implicated in brain accumulation of lipids and increased ceramide in the CSF of HIV/neuroAIDS subjects (Haughey et al., 2004; Farooqui et al., 2007). CSF levels of sphingomyelin, ceramide, and sterol species have been studied and compared with performance on standard neurological tests in 31 PWH (80% on ART; Mielke et al., 2010). Greater sphingomyelin/ceramide ratios for acyl chain lengths of C16:0, C18:0, C22:0, and C24:0 were associated with poorer performance on memory testing (Mielke et al., 2010). Moreover, higher sphingomyelin:cholesterol ratios were found in PWH with lower scores on the Rey Auditory Verbal Learning Test trail 5 and delayed recall tests (Mielke et al., 2010). Ceramide C16:0 and ceramide C22:0 levels were also evaluated in a prospective double-blind trial, in which PWH were treated with fluconazole, paroxetine or placebo. The study failed to prove a significant improvement in plasma and CSF lipid levels. However, in PWH treated with paroxetine, plasma ceramide C22:0 levels were reduced more than in those treated with placebo and also a cognitive improvement was noticed in the same group, although not consistent across all cognitive domains (Sacktor et al., 2018). In conclusion, data on lipid biomarkers trend in the course of suppressive ART are still lacking, and little is known on the potential use for diagnosis and follow-up of aviremic PWH.

### Extracellular Vesicles

Extracellular vesicles (EVs) are generated from most cell types and released into blood and CSF to carry and deliver cellular products to other neighboring or distant cells, including proteins, lipids, and nucleic acids (Thery et al., 2002; Rashed et al., 2017), but also, in the course of viral infection, viral proteins (Kadiu et al., 2012) and pro- or anti-inflammatory factors (Li et al., 2013). EVs can be classified as exosomes (50–150 nm, originate from multivesicular bodies) or microvesicles (200 nm to 1 μm, originate from plasma membrane), depending on their origin and particle size, and have been studied as possible markers of many neurologic disorders and neuroinflammatory and neurodegenerative diseases (Gupta and Pulliam, 2014; Coleman and Hill, 2015; Madison and Okeoma, 2015; Welton et al., 2017). Few studies have characterized their role and function in the course of HIV infection (Sun et al., 2017; Chettimada et al., 2018; Guha et al., 2019).

In a work including HIV-uninfected and HIV-infected subjects (67% aviremic), CSF EVs were more abundant in HIV-infected compared to HIV-uninfected subjects, regardless of NCI status (*p* < 0.0001) and were more abundant in NCI (*p* = 0.04 and *p* = 0.011 for EVs and CSF, respectively) or HAND (*p* < 0.0001 and *p* = 0.02 for EVs and CSF, respectively) compared to unimpaired HIV-infected subjects (Guha et al., 2019). CSF EV concentrations also showed a positive correlation with NFL levels, while no correlations were found with S100B and neopterin (Guha et al., 2019). Although the study of EV seems promising, evidence to support their use in clinical practice is still missing.

### Wnt Pathway

Wnts are a family of 19 highly conserved, small secreted glycoproteins that bind to the seven-transmembrane Frizzeled receptors and its co-receptor, LDL receptor-related proteins 5 and 6, to engage a signaling cascade that culminates in β-catenin-dependent or -independent signaling. The Wnt pathway plays a critical role in cell communication, differentiation, and survival (Al-Harthi, 2012; Clevers and Nusse, 2012), and its dysregulation has been linked to neurodegenerative diseases, including Alzheimer’s disease, Parkinson’s disease, and amyotrophic lateral sclerosis (Al-Harthi, 2012; Purro et al., 2014). To date, few data are available on the signaling and regulation of Wnt pathway in the course of HIV infection and HAND. Previous studies found that Wnt/β-catenin was a restriction factor for HIV in astrocytes (Li et al., 2011; Narasipura et al., 2012; Richards et al., 2015), and inhibition of Wnt/β-catenin signaling influenced the glutamate uptake and metabolism in astrocytes (Lutgen et al., 2016). The Dickkopf-related protein 1 (DKK1) is a secreted soluble antagonist of the Wnt pathway that induces the rapid disassembly of synapses in mature neurons, and it mediates synaptic loss induced by Aβ (Purro et al., 2012). In HIV-infected subjects, DKK1 levels did differ with NCI (mean 813 pg/ml vs. 549 pg/ml, *p* < 0.05), particularly in PWH taking ART with HIV RNA levels ≤50 c/ml (Yu et al., 2017). Even if studies are still lacking in HIV, Wnt pathway regulates synaptic transmission and plasticity, and its study could provide a new understanding of some neuropathological processes in the course of HAND.

### Mitochondrial DNA

Copy number of mtDNA in the brain and mitochondrial damage and mtDNA copy number within brain tissue have been correlated with a variety of neurodegenerative pathologies and aging. Moreover, considering the similarities of mtDNA with bacterial genomes, it acts as a “damage-associated molecular pattern” molecule, triggering toll-like receptor-9 activation and consequent inflammation. For all these reasons, quantity of mtDNA in CSF recently emerged as a biomarker of mitochondrial alteration and has been correlated with brain inflammation. Pérez-Santiago et al. (2016) first described an association between mtDNA levels in CSF and neurocognitive deficits in a group of 28 HIV+ subjects, most of which were not virologically suppressed neither in plasma nor in CSF at time of this cross-sectional analysis. Interestingly, a strong association was demonstrated between mtDNA in CSF and inflammatory markers, specifically IP-10 in CSF and Monocyte Chemo-Attractant Protein-1 (MCP-1) in plasma, in individuals with NCI, thus leading to the hypothesis that PWH with NCI may have inflammatory responses that differ from PWH without NCI and may also respond differently to the presence of mtDNA. A similar analysis was performed in the CHARTER population to determine the relationship between CSF mtDNA with CSF inflammatory markers, angiogenesis, iron transport, and HAND (Mehta et al., 2017). Interestingly, Mehta et al. (2017) found higher cell-free mtDNA levels in untreated PWH with detectable CSF HIV RNA. Even if these markers did not distinguish between PWH with or without NCI, mtDNA levels were higher in PWH with mild NCI as compared to asymptomatic subjects. There was also an association between CSF mtDNA and levels of interleukin-6 (IL-6) within the PWH group, even after adjustment for HIV RNA and CSF WBC. Moreover, levels of mtDNA correlated with lower levels of ceruloplasmin and transferrin, and the angiogenesis marker VEGF, after adjusting for the presence of HIV RNA and WBCs in CSF.

In a subsequent wok, Pérez-Santiago et al. (2017) analyzed the association between CSF, inflammation, and neurocognitive performance in PWH on long-term ART with persistent viral suppression in plasma. In this work, cell-free mtDNA in CSF samples has been inversely associated with peripheral and CSF inflammation [MCP-1 in CSF, and tumor necrosis factor-α (TNF-α) and IL-8 in plasma] under virologically effective ART (also after adjustment for past AIDS diagnosis). Indeed, contrary to the previous study, in this cohort of virally suppressed individuals, higher cell-free mtDNA levels within CSF supernatant were associated with better neurocognitive performance, as measured by the summary *T* score. Additionally, lower mtDNA in the CSF correlated with higher levels of NFL. Thus, in ART-treated PWH, a disequilibrium in cellular and mitochondrial function, similar to other neurodegenerative diseases such as Alzheimer’s disease and Parkinson’s disease, is recognized, which leads to mtDNA depletion, neuronal damage, and worse neurocognitive outcomes. Well-defined longitudinal studies will be needed to clarify the relationship between free mtDNA and neurocognitive outcomes during HIV suppression.

Another study approach focused on mtDNA is to define mitochondrial haplogroups, which have been shown to affect a range of HIV disease characteristics, including those potentially related to inflammation. Indeed, *in vitro* studies have shown that European mitochondrial haplogroups differ in expression and methylation of inflammation pathway genes. An analysis performed by Samuels et al. (2016) on participants from the CHARTER study who had genetic data and CSF samples showed no significant associations of any of the four measured CSF cytokines (IL-6, IL-8, IP-10, and TNF-α) with mitochondrial haplogroup in participants of African or Hispanic ancestry. However, in the subgroup of participants of European ancestry with suppressed plasmatic HIV-RNA viremia, the common haplogroup H had significantly lower CSF TNF-α levels (Samuels et al., 2016).

## MARKERS OF INFLAMMATION

During ART-mediated viral suppression, the dominant hypothesis for ongoing brain dysfunction has been linked to persistent inflammation. Following the introduction of ART, a sharp decrease of systemic inflammation has been demonstrated but not with resolution to normal levels. We summarized increasing evidence of the association between markers of inflammation and NCI (Table 2).

**Table 2:** Biomarkers of inflammation and correlation with NCI in people living with HIV.

Biomarker	Body fluid	Findings	Research gaps	Reference
MCP-1	CSF	Shown to correlate with change in NCI performance in mostly treated PWH; found to be higher in PWH with NCI compared to non-NCI; shown to be higher than plasma MCP-1 in PWH with NCI; elevated at baseline in HIV suppressed individuals with NCI switching therapy.	Lack of randomized trials; Lack of strong longitudinal data; Lack of studies conducted on large numbers of HIV suppressed individuals.	Marcotte et al. (2013), Yuan et al. (2013), Tiraboschi et al. (2015) and Yuan et al. (2015)
	PLASMA	MRI studies show correlation with subcortical injury; shown to be lower than CSF MCP-1 in PWH with NCI and shown not to correlate with NCI.	Inconclusive data; Lack of randomized trials; Lack of studies conducted on large numbers of HIV suppressed individuals.	Ragin et al. (2006)
TNF alpha	CSF	Shown to decrease in 24 weeks in suppressed PWH with NCI after switch to an ART regimen with high neuropenetration; Levels found to be slightly lower in PWH having started ART early compared to late ART.	Lack of randomized trials; Limited population numbers.	Tiraboschi et al. (2015) and Oliveira et al. (2017)
IL-6	CSF	Levels found to be slightly lower in PWH having started ART early compared to late ART; showed by one study to correlate to a reduced likelihood of NCI in PWH, both naïve and on ART.	Inconclusive data; Lack of randomized trials; Lack of longitudinal studies; Lack of studies conducted on large numbers of HIV suppressed individuals	Oliveira et al. (2017) and Kallianpur et al. (2018)
IP-10/CXCL10	CSF	Found to be higher in PWH with NCI compared to non-NCI; higher levels in ART-treated PWH with NCI than in untreated PWH with NCI.	Lack of randomized trials; Lack of longitudinal studies.	Yuan et al. (2013) and Yuan et al. (2015)
	PLASMA	Found to be higher in PWH with NCI compared to non-NCI; plasma levels shown to decrease in a study investigating the use of paroxetine and fluconazole for the treatment of HAND.	Lack of longitudinal studies; viability as a biomarker in clinical practice has yet to be tested.	Yuan et al. (2015) and Sacktor et al. (2018)
IL-8 or CXCL-8	CSF	Found to be higher in PWH with NCI compared to non-NCI; shown to be higher than plasma IL-8 in PWH with NCI; CSF levels shown to increase in a study investigating the use of paroxetine and fluconazole for the treatment of HAND.	Inconclusive data; Lack of longitudinal studies; Lack of studies conducted on large numbers of HIV suppressed individuals	Yuan et al. (2013) and Sacktor et al. (2018)
IFN-α	CSF	Found to negatively correlate with several individual and composite neuropsychological tests, despite lack of correlation with treatment status.	Lack of randomized trials; Lack of strong longitudinal data; Lack of studies conducted on large numbers of HIV suppressed individuals.	Anderson et al. (2017)
IL-16	Serum	Found to be associated with slower processing speed.	Lack of randomized trials; Lack of strong longitudinal data; Lack of studies conducted on large numbers of HIV suppressed individuals.	Okafor et al. (2017)
ICAM5	CSF	Found to be higher in PWH with NCI compared to non-NCI, and to correlate with disease progression in a small number of PWH.	Lack of randomized trials; Lack of strong longitudinal data; Lack of studies conducted on large numbers of HIV suppressed individuals.	Yuan et al. (2017)
	Plasma	Found to be higher in PWH with NCI compared to non-NCI, and to correlate with disease progression in a small number of subjects.		
Microbial translocation markers	PLASMA	LPS levels found to correlate with HAND in a subset of HCV-positive participants; LPS found to be a significant predictor of lower processing speed in ART suppressed heavy drinkers; BDG levels found to correlate to worse neurocognitive performance.	Lack of randomized trials; Lack of strong longitudinal data; Lack of studies conducted on large numbers of HIV suppressed individuals.	Vassallo et al. (2013), Hoenigl et al. (2016) and Monnig et al. (2017)
Growth factors	CSF	G-CSF found to be higher in PWH with NCI compared to non-NCI.	Lack of randomized trials; Lack of strong longitudinal data; Lack of studies conducted on large numbers of HIV suppressed individuals.	Yuan et al. (2015)
	PLASMA	CSF VEGF showed to have borderline association with GDS-defined NCI; ceruloplasmin and CSF haptoglobin levels associated to likelihood to develop NCI; G-CSF found to be higher in PWH with NCI compared to non-NCI.		Yuan et al. (2015) and Kallianpur et al. (2018)

*Abbreviations: NCI, neurocognitive impairment; ART, antiretroviral therapy; CSF, cerebrospinal fluid; GDS, global deficit score; PWH, people with HIV*.

### Monocyte Chemo-Attractant Protein (MCP-1)

Among the most studied biomarkers of inflammation, MCP-1 is a chemokine produced by a number of cells constitutively or after oxidative stress (including activated microglia and astrocytes) and, by regulating migration and infiltration of monocytes/macrophages, contributes to neuroinflammation and injury (Deshmane et al., 2009; Rahimian and He, 2016). A work from 2006 investigated the relationship between biomarkers and MRI diffusion tensor imaging measurements of centrum semiovale, caudate, and putamen, which have been shown to correlate with cognitive status. In 11 ART-treated HIV-infected subjects, plasma MCP-1 levels correlated with subcortical injury (Ragin et al., 2006). A 2013 work aimed at investigating biomarkers within 98 HIV-infected individuals categorized according to neurocognitive performance (including “stably impaired” and “worsening”), of which 73% of PWH were on ART and 54% were virally suppressed. Linear regression identified that, among markers, only MCP-1 in CSF was associated with neurocognitive change. Models constructed with the aim of diagnosis showed that a combination of MCP-1 and TNF-α allowed classifying 100% of the subjects with stable impairment (Marcotte et al., 2013). In a 2013 preliminary study, Yuan et al. (2013) measured cytokine levels in the CSF of 107 HIV-infected PWH (43% of whom were on ART) with and without NCI through quantification bioassays. Cases with NCI demonstrated significantly higher levels of MCP-1 (along with IL-8 and IP-10; Yuan et al., 2013). In 2015, Yuan et al. (2015) compared cytokine levels in paired CSF and plasma samples from 85 HIV+ individuals (43% of whom were on ART) with or without NCI. The expression of MCP-1 was significantly higher in CSF compared to plasma, and more importantly, CSF MCP-1 was significantly higher among subjects with NCI, while plasma MCP-1 did not correlate with cognitive impairment (Yuan et al., 2015). In a 2015 prospective, single-arm pilot study by Tiraboschi et al. (2015), 12 ART suppressed PWH with NCI on a regimen including TDF/FTC/EFV were switched to ABC/3TC/MVC. All participants showed elevated MCP-1 levels (along with neopterin) in CSF at baseline, although no significant change in MCP-1 levels was detected after week 24 from switch to the regimen with high neuropenetration (Tiraboschi et al., 2015). In summary, CSF MCP-1 showed promising correlation to NCI in multiple independent studies with distinct endpoints and designs. However, most studies encompassed both untreated and treated PWH (with some of the latter still being viremic); therefore, the role of CSF MCP-1 as a viable biomarker for NCI in ART-suppressed PWH is yet to be assessed.

### Tumor Necrosis Factor Alpha (TNF-α)

TNF-α is a proinflammatory cytokine that exerts homeostatic and pathophysiological roles in the CNS. TNF-α is released in large amounts by microglia in pathological conditions, and this production constitutes part of the neuroinflammatory response and has been associated with a number of neurological disorders (Olmos and Lladó, 2014). In the aforementioned 2015 prospective, single-arm pilot switch study by Tiraboschi et al. (2015), a statistically significant reduction in median TNF-α concentration in CSF was observed after a 24-week switch to an ART regimen with high neuropenetration; no significant differences were observed, taking other inflammatory markers into exam. Of note, median CSF HIV RNA decreased as well (all participants had suppressed plasma HIV RNA; Tiraboschi et al., 2015). In a study by Oliveira et al. (2017), paired blood and CSF samples were collected from 16 HIV-infected individuals on suppressive “early” or “late” ART. Median CSF TNF-α levels were slightly but significantly lower in individuals on early ART compared to those who had initiated ART later. No difference was detected among groups for blood TNF-α (Oliveira et al., 2017).

In conclusion, there is limited but promising evidence in favor of TNF-α being associated with inflammation due to uncontrolled/residual viremia. Further studies are warranted.

### Interleukin-6

IL-6, a mediator of the acute phase response, can be produced by astrocytes exposed to HIV (Nitkiewicz et al., 2017). Lower IL-6 levels in CSF have been observed in individuals who had started ART early compared to those who had initiated ART later in a work by Oliveira et al. (2017). No difference was detected among groups for plasma IL-6 concentration. These data supported the concept that early ART initiation reduces at least some inflammation mediators in CSF. A possible effect of IL-6 on CSF HIV DNA molecular diversity was also explored but no mediation effect was observed (Oliveira et al., 2017). In a 2017 work investigating the relationship between CSF levels of proteins involved in iron transport and/or angiogenesis and neuropsychiatric impairment in 405 HIV-positive individuals (73% of subjects on ART, 46% of PWH were virally suppressed), CSF levels of IL-6 were not found to correlate with HAND. Of note, CSF IL-6 was shown to correlate to a reduced likelihood of impairment in 135 patients with mild-moderate comorbidity (Kallianpur et al., 2018). Despite promising reports, data concerning a possible role of CSF IL-6 in NCI of PWH are inconsistent. Further studies are needed to expand the current knowledge.

### Interferon-γ-Inducible Protein (IP-10 or CXCL10)

IP-10 is a CXC or α-chemokine that acts on its receptor, CXCR3, to attract activated T cells, NK cells, and blood monocytes. A potent chemoattractant, it has been proposed to enhance retroviral infection and mediate neuronal injury. CSF IP-10 levels are known to correlate with CSF HIV-RNA and to decrease in subjects starting ART (Cinque et al., 2005). In the aforementioned 2013 preliminary study by Yuan et al. (2013) on 107 HIV-infected individuals, significantly higher levels of IP-10 (along with IL-8 and MCP-1) were found in the CSF of individuals with NCI compared to those without NCI. Interestingly, IP-10 was the only marker found to be associated with ART treatment, as CSF IP-10 was found to be higher in ART-treated PWH with NCI than in untreated PWH with NCI (Yuan et al., 2013). Again, in 2015, Yuan et al. (2015) compared cytokine levels in paired CSF and plasma samples from 85 HIV-infected individuals with or without NCI. Both CSF and plasma IP-10 were found to correlate with each other in the PWH cohort (Yuan et al., 2015). In 2018, Sacktor et al. (2018) published the results of a double-blind, placebo-controlled trial evaluating paroxetine and fluconazole for the treatment of HAND. Paroxetine was associated with improvement in a summary neuropsychological test measure, and in some—but not all—distinct neuropsychological tests. The authors investigated changes in biomarkers of cellular stress, inflammation, and neuronal injury over 24 weeks in response to treatment. Both paroxetine treatment alone and combined paroxetine/fluconazole treatment showed a decrease in plasma IP-10 levels compared to placebo (Sacktor et al., 2018).

In conclusion, a small number of independent studies with distinct designs and endpoints correlate IP-10 to NCI, interestingly, both as a plasma and CSF marker. IP-10 seems to be a reliable marker for NCI during ART, and correlation to a novel treatment for HAND has been described. However, viability as a biomarker in clinical practice has yet to be tested.

### Interleukin-8 (IL-8 or CXCL-8)

Produced by macrophages and other cell types, IL-8 is the primary cytokine involved in neutrophil chemotaxis. In the cited 2013 and 2015 studies by Yuan et al. (2013, 2015). CSF levels of IL-8 were significantly higher in HIV-infected individuals with NCI compared to those without NCI. IL-8 levels were significantly higher in CSF than plasma levels (Yuan et al., 2013, 2015). On the other hand, while the 2018 paroxetine/fluconazole trial by Sacktor et al. (2018) suggest a beneficial effect of paroxetine on HAND, both paroxetine treatment alone and combined paroxetine/fluconazole treatment showed an increase in CSF IL-8/CXCL-8 levels compared to placebo. Therefore, despite promising reports, data concerning a possible role of CSF IL-8 in NCI of ART suppressed PWH is inconsistent.

### Interferon Alpha

A renowned antiviral cytokine, interferon α (IFNα) has been found to be elevated in CSF of individuals with advanced HAD since the pre-combined ART era.

A 2016 cross-sectional study investigated the association between IFNα and neurocognitive performance, measured by an eight-test neuropsychological battery. Of 15 PWH, 60% were ART-experienced and 20% had undetectable viral load; CSF and plasma IFNα levels did not differ between treated and untreated or suppressed and non-suppressed PWH. CSF IFNα was found to negatively correlate with three individual tests and the composite test. Additionally, CSF IFNα correlated strongly with CSF NFL. Plasma IFNα did not show a significant correlation to cognitive performance (Anderson et al., 2017). While these results strongly suggest that CSF IFNα continues to play a role in HAND pathogenesis during the cART era, they pertain to a single study. Further works are needed to establish the correlation of IFNα and NCI in ART-suppressed PWH.

### Interleukin-16

IL-16 is a pleiotropic cytokine acting as a chemoattractive and modulating factor of T cell activation. In a 2017 work aimed at determining the relationship between body mass index (BMI), HIV-associated NCI, and the potential mediating effects of inflammatory cytokines, 90 HIV-infected PWH (86.7% of whom were virally suppressed) were evaluated. Serum concentrations of IL-16 were significantly associated with slower processing speed, independently of BMI (Okafor et al., 2017). None of the other studies taken into consideration in the present review showed a significant correlation between IL-16 and NCI. Further studies are needed to establish such a correlation in ART-suppressed PWH.

### Intercellular Adhesion Molecule-5

Intercellular adhesion molecule-5 (ICAM5) is an ICAM expressed on neurons that may inhibit CNS T cell activation. Elevated levels of ICAM5 have been previously found in patients with brain injury (Guo et al., 2000; Di Battista et al., 2015).

Only one previous study, according to the selection criteria chosen for the present review article, addressed changes in ICAM5 in the HIV-infected population (Yuan et al., 2017). In that study, higher ICAM5 concentrations were found in PWH with NCI compared to PWH without impairment. Plasma and CSF ICAM5 levels showed a significant correlation to each other, and plasma ICAM5 significantly correlated with CSF S100B (Yuan et al., 2017). Moreover, high concentrations of plasma and CSF ICAM5 were found in PWH who developed NCI while levels did not change in individuals who did not develop any form of impairment. The change of plasma ICAM5 levels always corresponded with that in CSF (Yuan et al., 2017). Future studies will clarify the applicability of these preliminary findings in the HIV-infected population.

### Eotaxin

Eotaxin is a chemokine that acts as a potent eosinophil chemoattractant and that, according to recent studies, may also contribute to degenerative processes in the CNS (Huber et al., 2018). However, only one study, among those selected for the present review article, addressed the evaluation of this biomarker, failing to determine any significant findings. In 85 HIV-infected subjects (43% of whom were on ART), eotaxin levels did not correlate with the diagnosis of NCI, neither in CSF nor in plasma (Yuan et al., 2015).

### Microbial Translocation Markers

Chronic inflammation after ART introduction has been associated with microbial translocation with bacterial and fungal antigens across damaged gastrointestinal tract to drive systemic and CNS inflammation.

A component of the cell wall of gram-negative bacteria, lipopolysaccharide (LPS), can reach high plasma levels with translocation of microbial products across the intestinal mucosa into the peripheral circulation. LPS is suspected to trigger monocyte activation and to increase trafficking of infected cells into the brain (Epple et al., 2009). However, LPS measure is not easy to perform, as LPS assays are problematic because of blocking factors in bio-fluids and different types of LPS. Therefore, LPS cannot be measured in the standard ELISA format, and results of bioassays provide measures that are not fully comparable between different sites. In a work by Vassallo et al. (2013), LPS plasma levels were compared between 179 PWH with HAND and those with no HAND (87% of subjects on ART and 67% virally suppressed). A clear association was found between LPS levels and HAND in a subset of HCV-positive participants, while the association was non-significant among the HCV-negative group (LPS had previously been shown to be elevated in hepatitis C co-infection; Vassallo et al., 2013). LPS, along with its ligand sCD14, was also found to be a significant predictor of lower processing speed in a 2017 randomized clinical trial focused on alcohol intervention on ART-suppressed heavy drinkers living with HIV (Monnig et al., 2017).

1,3-β-D-glucan (BDG) is a component of most fungal cell walls and therefore is thought to be another useful indicator of gut mucosal barrier impairment. In a 2016 cross-sectional cohort study, levels of BDG were measured in plasma and CSF in 21 adults with acute/early HIV infection, started on ART during the earliest phase of infection with HIV RNA suppression. Higher plasma BDG levels were significantly related to higher GDSs, reflecting worse neurocognitive performance. Interestingly, CSF BDG was only found to be elevated in two individuals, those with the highest GDSs (Hoenigl et al., 2016).

In light of the wide body of work supporting the gut–brain barrier as a novel target in the ART era, LPS is a promising biomarker. LPS correlates with processing speed at least in a very specific subset of HIV-positive, ART-suppressed PWH. However, HCV co-infection represents a significant confounding factor and further studies on LPS and BDG are warranted in ART-suppressed PWH.

### Growth Factors

#### Iron Transporters and VEGF

Iron is necessary for mitochondrial function, and its transport is thought to influence immune activation and angiogenesis (Cherayil, 2010; Saghiri et al., 2015); iron availability can be both related to HAND and neurodegenerative diseases (Bhatia and Chow, 2016; Edén et al., 2016). Altered angiogenesis, in particular, may lead to disruption of blood-brain barrier integrity in PWH, with consequent possibility of migration of activated immune cells into the CNS and development of inflammation and infection (Nightingale et al., 2014). In one single study satisfying inclusion criteria for the present review, VEGF levels and iron transporters (i.e., ceruloplasmin and aptoglobin) have been studied in relation to NCI in HIV (Kallianpur et al., 2018). Elevated CSF VEGF levels were associated with higher GDSs and with NCI, defined by GDS (Kallianpur et al., 2018). Moreover, aviremic PWH without neurological comorbidities and with higher ceruloplasmin levels were more likely to have GDS impairment. The same results were also found in PWH with higher CSF haptoglobin levels (Kallianpur et al., 2018). Despite those preliminary results, the authors of the study highlighted that the associations they found were not adjusted for multiple testing and required thus replication and further investigation (Kallianpur et al., 2018).

#### Fibroblast Growth Factors

Fibroblast growth factors (FGFs) are implicated in brain development and in neuroprotective functions. An alteration of FGF levels in CSF may be linked to neuronal injury and has been previously described in the course of amyotrophic lateral sclerosis (Johansson et al., 2003), moyamoya (Yoshimoto et al., 1997), and Alzheimer’s disease (Mashayekhi et al., 2010). FGF levels in CSF have been poorly studied in humans and, in particular, in the setting of HIV-infected subjects (Bharti et al., 2016). However, one study performed among 100 PWH (37% of whom were aviremic) failed to prove a consistent correlation between FGF-2 levels and cognitive disorders, while finding a correlation between lower FGF-1 levels and NCI (Bharti et al., 2016). The body of evidence regarding this marker in the clinical practice or in studies focused on NCI in the course of HIV is thus still scarce.

#### Granulocyte Colony-Stimulating Factor

Granulocyte colony-stimulating factor (G-CSF) is a hematopoietic growth factor that stimulates proliferation and differentiation of myeloid cells. However, G-CSF and its receptor are also expressed by neurons in many brain regions and recent studies suggested its role as a neurotrophic factor, as G-CSF has an anti-apoptotic function and stimulates neuronal differentiation in the brain (Schneider et al., 2005). In the context of HIV-related NCI, few studies have evaluated G-CSF levels. In a previous study performed in 85 HIV-infected subjects (43% of participants were on ART; Yuan et al., 2015), G-CSF levels did not differ significantly in plasma and CSF, indicating a possible reliability of blood measurement of this marker without the need of lumbar puncture. However, G-CSF levels were significantly higher in PWH with NCI compared to those who had no impairment, in both CSF (*p* = 0.0079) and plasma (*p* = 0.0191; Yuan et al., 2015). In another study evaluating a total of 107 CSF samples from PWH, G-CSF levels in the CSF were not different between ART-treated and untreated PWH but were higher in PWH with impaired cognition (Yuan et al., 2013).

Those preliminary results may suggest specificity in G-CSF levels in identifying PWH with HAND, also in the context of ART. However, no longitudinal studies are available to date and the evidence for its use remains very scarce.

## Immune Activation Markers

### T Cell Activation

Considering the emerging role of inflammation as a contributing factor in neurological damage in the setting of ART-mediated virological suppression, well-characterized immune activation drivers have been explored as potential biomarkers of NCI during ART. Low CD4/CD8 ratio despite suppressive ART has been linked to immune activation and used to identify PWH with accelerated aging at risks of clinical events, including NCI. By evaluating the association of LPS levels with NCI, Vassallo et al. (2013) also considered CD4/CD8 ratio, which was significantly associated with NCI in HCV-negative subjects in univariate analysis. However, after considering age, proviral DNA, CD8 T cell count, and CD4/CD8 ratio in a logistic regression model, only age and proviral HIV-DNA resulted independently associated to NCI. In a subsequent work, Vassallo et al. (2013) explored the association between CD4/CD8 ratio, T-lymphocyte activation, and NCI in a large cohort (200 PWH) of virologically suppressed individuals on ART. They found that in ART-treated PWH on stable therapy for at least 3 years with virological suppression, a CD4/CD8 <1 resulted in an independent risk factor for symptomatic HAND (Vassallo et al., 2013). Moreover, a significant correlation between CD4/CD8 ratio and T-cell activation (measured by the number of CD4+CD38+HLADR+ T lymphocytes) was found, contributing to the evidence that CD4/CD8 ratio can be considered as a marker of chronic immune activation. A similar analysis was performed in MSM (men who have sex with men) Neurocog Study, in which 200 HIV-infected MSM PWH were screened for NCI and, after excluding people suffering from anxiety, depression or previous diagnosis of mood disorders, 20 were identified as potentially having NCI (Rawson et al., 2015). In this study, no significant difference in current, nadir or peak CD4 and CD8 counts, CD4/CD8 ratios, CD4/CD8 ratio inversion (<1), plasma viral load detectability, and peak plasma viral load were identified between subjects with NCI and the control group. Thus, until now, no definitive demonstration has been reached to define any significant benefit in monitoring CD8 T cells or CD4/CD8 T cell ratio inversion as an indicator of NCI.

Recently, Merlini and coauthors evaluated the effects of 12 months of virally suppressive ART on the peripheral and CSF pro-inflammatory milieu (Merlini et al., 2018). As expected, they found a decline in both plasma and CSF viral replication after 12 months of ART, and a simultaneous reduction in peripheral and CSF immune activation, measured by CD38+CD8+ cells, sCD14, IL-6, MCP-1, and IP-10. Interestingly, subjects with high pre-ART CSF/plasma HIV-RNA ratio maintained a skewed T cell homeostasis toward more effector/exhausted phenotype after ART introduction. In contrast, HIV people with low CSF/plasma HIV-RNA at baseline displayed a trend toward recovered peripheral CD4+ and CD8+ T cells phenotypes, thus suggesting that a lower pre-ART CSF viral burden and a subsequent viral suppression can prevent CNS invasion by HIV and consequent detrimental immune activation.

### Monocyte/Macrophage Activation

Considering the role of the monocyte–macrophage lineage in the HIV-associated CNS disease, activation specifically involving these cells has been studied as a potential marker of neurocognitive damage during ART-mediated viral suppression.

#### Neopterin

Neopterin is a metabolite of guanosine triphosphate catabolism, and its increase in CSF is considered a marker of macrophage and microglial activation (Rahimian and He, 2016) and an independent predictor of CSF NFL levels (Jessen Krut et al., 2014). CSF neopterin levels are usually high in PWH who do not receive ART (Fuchs et al., 1989) and may decrease after ART initiation, possibly in parallel with improvement of neurocognitive function in the course of ART (Andersson et al., 2006). However, in certain individuals, some levels of immune activation may persist in the CNS also after the achievement of undetectable HIV-RNA levels, and the concentrations of CSF neopterin remain elevated for a long time also after having started ART (Edén et al., 2007; Yilmaz et al., 2013). In ART-treated PWH, neopterin level correlates with white matter hyperintensities that are in turn associated with neurological complications, including cognitive impairment (ρ = 0.321, *p* = 0.064; Trentalange et al., 2018). However, the direct correlation between neopterin levels and NCI in HIV-infected aviremic subjects is not univocal. In a recent work including 112 subjects (67% aviremic), neopterin was not an independent biomarker of baseline NCI status in multivariable linear regression models, adjusted for age, race, and plasma viral load (Guha et al., 2019). These findings corroborated the results of two previous studies, in which no correlation was found between CSF neopterin and GDS (Bharti et al., 2016; Pérez-Santiago et al., 2016). Both studies were limited by a relatively small sample size, and a large part of the study population had detectable HIV viremia. However, CSF neopterin levels were not found to be significantly different between neurocognitively normal, ANI, and MND PWH in another study conducted on a population of aviremic PWH (*n* = 37; Burdo et al., 2013). On the other hand, in that same study, higher levels of neopterin were found in PWH who were more impaired by GDS criteria than in those without cognitive impairment, and higher levels were also found in people with an executive domain impairment compared to others (Burdo et al., 2013). Similar results were reported in another recent study performed in aviremic PWH, in which CSF neopterin was higher in aviremic PWH with NCI compared to others (*p* = 0.04; Edén et al., 2016).

However, a study performed on 99 aviremic PWH followed up longitudinally did not prove a difference in the proportion of abnormal neopterin levels between PWH with or without a decline in the neurocognitive function (Edén et al., 2016). In another longitudinal evaluation, after persistent undetectable viremia, CSF neopterin at second visit was significantly higher than at first visit after a median of 16 months, despite ART use and long-term viral suppression (*p* = 0.05, paired *t*-test; Burdo et al., 2013). Furthermore, no changes in neopterin levels were found in a longitudinal study evaluating ART switch to regimens with enhanced CNS penetrability (Tiraboschi et al., 2015), neither in PWH initiating paroxetine in a double-blind trial, in which paroxetin use correlated with improvement in neurocognitive function (Sacktor et al., 2018). The correlation of CSF neopterin levels and scores of different neuropsychological tests has also been evaluated in aviremic PWH. Higher levels of neopterin were correlated with poorer results on forward Corsi Block Tapping Test (*r* = −0.474; *p* = 0.004), backward Corsi Block Tapping Test (*r* = −0.468; *p* = 0.005), forward Digit test (*r* = −0.480; *p* = 0.004), Verbal Fluency test (*r* = −0.361; *p* = 0.033), Raven’s Standard Progressive Matrices test (*r* = −0.308; *p* = 0.071), the time estimation during the Test of Weights and Measures Estimation (*r* = −0.295; *p* = 0.085) and the Test of Weights and Measures total score (*r* = −0.294; *p* = 0.087; Ceccarelli et al., 2017). In conclusion, while neopterin CSF levels might be considered a reliable marker of macrophage and microglial activation and a predictor of worse performance in some cognitive domains, its use in the longitudinal follow-up of aviremic PWH is not sustained by current evidence. Further studies, possibly combining the evaluation of many biomarkers of immune activation and neuronal injury, could help improve its utility in the definition and diagnosis of neurologic disorders in the course of HIV.

#### Soluble CD14

Soluble CD14 (sCD14) is the soluble form of the monocyte LPS receptor, cleaved and released from the membrane after monocyte activation (McGuire et al., 2015). In a 2011 study by Lyons et al. (2011), relationships between plasma sCD14, CCL2, and LPS levels and neurocognitive test scores were investigated in 97 HIV-infected subjects. Plasma sCD14 levels were higher in subjects with test scores indicating global impairment, particularly in attention and learning domains, regardless of HAND diagnosis. The authors suggest such results imply the involvement of cortical and limbic pathways by inflammatory processes in the cART era. Of note, individuals in the study population were on ART in 74% cases, of which 39% had undetectable plasma HIV RNA (Lyons et al., 2011). In 2017, a secondary analysis on a randomized clinical trial focused on alcohol intervention was published by Monnig et al. (2017) investigating the relationship between the gut–brain axis, HIV infection, and alcohol consumption. Blood samples and cognitive scores were obtained at baseline and 3-month follow-up of the trial from 21 HIV-positive, ART-suppressed, heavy drinkers. sCD14 (along with LPS) was found to be a significant predictor of lower processing speed (Monnig et al., 2017). In a 2018 work evaluating MRI white matter hyperintensity (WMH) in naive compared to treated HIV-infected individuals (*n* = 107), plasma sCD14 levels were weakly associated with WMH score; however, no consistent associations between plasma biomarkers, CSF biomarkers, and WMH scores were found. Of note, sCD14 levels were lower in treated PWH compared to naive individuals (Trentalange et al., 2018). In conclusion, there is evidence that plasma sCD14, a well-studied monocyte activation marker, correlates with processing speed at least in a very specific subset of ART-suppressed PWH. Promising data suggest a correlation with global impairment and MRI signal, but new studies are warranted involving aviremic PWH exclusively.

#### Soluble CD163

One of the most promising pathways, CD163, is released from macrophage surface and shed as soluble CD163 (sCD163) following activation and differentiation of monocyte and macrophages. It has been hypothesized that virologically suppressed PWH would show persistent activation of monocytes, which could be assessed by measuring sCD163 in blood, which would correlate with HAND. A 2013 longitudinal study by Burdo et al. (2013) on 34 ART-suppressed individuals showed that PWH with NCI by GDS metrics had higher plasma sCD163 than those who were not impaired. Interestingly, CSF sCD163 did not correlate to GDS. Such results were discussed to be consistent with persistent monocyte/macrophage activation in neurophysiologically impaired HIV-infected individuals despite virally suppressive ART (Burdo et al., 2013). In a 2015 study, the relationship between cell-free mtDNA in CSF and neurocognitive performance during HIV infection, CSF sCD163 was the sole soluble inflammatory biomarker whose levels trended toward being higher in PWH with NCI compared to those without NCI. Of note, only 45% PWH were on ART (33% with viremia below the threshold of detectability; Pérez-Santiago et al., 2016). Bryant et al. (2017) conducted a study in 2017 evaluating sCD163 levels in ante mortem plasma (*n* = 54) and CSF (*n* = 32) samples from 74 HIV-infected participants who donated their brains to research at autopsy. Higher plasma sCD163 was found to be associated with greater synaptodendritic damage and microglial activation in cortical and subcortical brain regions at autopsy. Despite this, interestingly, plasma sCD163 was showed not to have any correlation to HAND diagnosis and neuropsychological test performance. On another note, CSF sCD163 was not associated with any histological feature (Bryant et al., 2017). In conclusion, a group of diverse studies suggest that plasma sCD163 correlates with NCI in virologically suppressed ART-treated individuals and to neural damage in PWH, regardless of symptoms, and CSF sCD163 tends to be higher in PWH with NCI. Such results, albeit partly inconsistent, are promising and warrant further investigation.

#### Monocyte Phenotypes

A number of studies focused on different monocyte phenotypes in blood that may drive HAND neuropathogenesis. Specifically, HIV infection has been correlated to an expansion of CD14+ monocytes expressing CD16+: CD14++CD16+ (intermediate) and CD14+CD16++ (nonclassic) monocytes, reaching variable levels depending on the stage of disease and use of ART. As CD16+ monocytes express higher levels of cell migration markers (i.e., CXCR5, CXCR1, and integrin CD11b) and transmigrate across the blood-brain barrier more efficiently than CD16− cells, it has been suggested that such cells can be involved HAND pathogenesis. In a recent study, a trend toward lower proportions of circulating intermediate monocytes in PWH who displayed a significant decline in memory performance during ART-mediated viral suppression was shown, suggesting an increase of transmigration of this cell subset in the CNS (Fabbiani et al., 2017). In addition, PWH with a significant decline in memory performance over time had lower expression of surface CD163 on the subset of intermediate monocytes/macrophages, corresponding to a higher release of sCD163 (Fabbiani et al., 2017).

As activated macrophages secrete soluble factors potentially toxic to neurons, some authors focused on the measurement of cytokines, proteases, and other factors produced by these cells after stimulation. Cathepsin B, a factor secreted by macrophages, has been described as a protease linked to neuronal apoptosis and inflammation. Moreover, cathepsin B, together with cystatins B and C, has been shown to be overexpressed by HIV-infected monocyte-derived macrophages (MDMs). In a study conducted on a population of mostly ART-treated (>80%) HIV women, Cantres-Rosario et al. (2013) showed a significantly higher proportion of intracellular cathepsin-B and cystatin B in CD14+ cells of HIV+ subjects with HAD compared to subjects without neurological impairment. Additional analyses on the same study evaluating cathepsin B levels and activity together with cystatin C levels on monocytes did not reveal any difference between HAD subjects and controls. Taken together, these studies suggest a role for monocyte subpopulation as candidate biomarkers and suggest new opportunities to target viral reservoirs within the CNS, thus reducing neuroinflammation, neuronal damage, and cognitive impairment.

## Omics Approach to Nci Markers Research

### Genomics

Considering that not all HIV-infected subjects develop some form of neurocognitive disorder, the study of genetic factors that could possibly predispose to neurologic disease has attracted increasing interest and some studies aimed at characterizing the genetic profile of HIV-infected individuals at increased risk for HAND. CCL3L1, a potent ligand for CCR5 receptor and chemoattractant for macrophages, displays significant copy number differences among different ethnic groups. In this regard, Gonzalez et al. (2005) reported that the risk of AIDS events, including NCI, was higher when CCL3L1 chemokine gene copy was lower than the average of the same ethnic group, together with CCR5 detrimental alleles. A subsequent study performed by Brown et al. (2012) confirmed previous data about differences in CCL3L1 median copy number between various ethnicities; however, CCL3L1 copy number was similar in patients infected with HIV who had any form of HAND and in those who did not. Levine et al. (2016) in a very comprehensive study quantified multiple histopathological markers, genotyped genes associated with risk of NCI, and measured HIV-RNA in brain tissues. In this multilevel analysis, they found that MAP2 and SYP, which are two markers of synaptodendritic integrity, showed the more robust correlation with HAND with global pre-mortem cognitive function and with HIV-RNA viral load in the same regions. Moreover, a concomitant increase of Aβ and Iba-1 (ionized calcium-binding adaptor molecule-1) was evidenced as HIV-RNA increased, suggesting dysfunctional protein clearance and neuroinflammation. Genetic markers that predicted histopathology included MIP1-α and DRD3 genotype that are predictors of Iba-1 immunoreactivity, IL1-α genotype that predicted GFAP (glial fibrillary acidic protein) immunoreactivity, and ApoE genotype that predicted Aβ immunoreactivity. These data seem to support a pathogenetic model in which CNS HIV replication represents one of the main drivers for neuroinflammation and abnormal clearance. As a consequence, synaptodendritic degeneration occurs, leading to NCI of proteins. Genetic polymorphisms in genes encoding cytokines and chemokines and neuronal protein clearance pathways could influence histopathological degeneration.

### Transcriptomics

Levine et al. (2013) in the last years applied a new research approach by examining the comprehensive gene expression of blood monocytes, with the aim of identifying the transcriptional changes possibly linked to HIV-associated NCI. Specifically aiming to describe peripheral molecular genetic mechanisms favoring the development of HAND, they firstly described that dysregulation of Kelch-like ECH-associated protein-1 (KEAP1), hypoxia upregulated-1, and IL-6 receptor, implicating oxidative stress, constituted a possible underlying pathogenic process. In a recent work published in 2018 (Quach et al., 2018), they further expanded these observations by validating the original findings in an independent sample of participants from the Multicenter AIDS Cohort Study (MACS) and determined whether gene expression changes evaluated at baseline could predict NCI at 2-years of follow-up. Contrary to what is expected, results from this new study did not replicate observation from their previous analysis and gene expression profiles at baseline were not predictive of neurocognitive decline in the following 2 years. Considering the inconsistency of these two observations, we have no evidence that gene expression profiles of monocytes can constitute a reliable biomarker of HAND.

### Proteomics and Metabolomics

Multiplex mass spectrometry-based approaches allow the analysis of multiple samples in a single experiment. MS techniques make it possible to study a multitude of proteins and their differential level of expression at the same time in the course of CNS diseases. PWH with HAND have been compared to PWH without HAND and to uninfected controls (Bora et al., 2014) using proteomics approach, studying 193 different proteins. A cut-off of 1.5-fold was used to define protein upregulation. Five proteins among those previously described as HIV-interacting proteins were found to be upregulated in PWH with HAND: endoplasmin, mitochondrial damage mediator-BH3-interacting domain death agonist, orosomucoid, APOE, and metalloproteinase inhibitor 2. On the contrary, using a cut-off of 0.6-fold to measure downregulation, peroxiredoxin-2 isoform, a ruvB-like 2 protein, had lower expression in HAND (Bora et al., 2014). Untargeted CSF metabolite profiling was also used with the aim of identifying an altered metabolic path that can be linked to HAND. Neurotransmitter production, mitochondrial function, oxidative stress, and metabolic waste were studied (Cassol et al., 2014). CSF metabolites of ART-treated PWH with HAND were compared to unimpaired patients with similar demographic characteristics. Despite the metabolite expression being similar in the two groups after multiple testing correction, recursive support vector machine (SVM) classification models identified eight metabolites (including glutamate, myo-inositol, beta-hydroxybutyric acid, 1,2-propanediol) capable of discriminating the presence of HAND with an accuracy that exceeded 85%. Glutamate and N-acetylaspartate of myo-inositol and ketone bodies (beta-hydroxybutyric acid, 1,2-propanediol) showed increased levels in HAND and also had a correlation with lower scores in neurocognitive tests, plasmatic markers of inflammation, and intrathecal interferon responses (Cassol et al., 2014). The profile of the biomarkers that have been delineated in this study highlights how, also in the absence of detectable HIV-RNA, an abnormal synthesis of neurotransmitters, glial activation, altered mitochondrial function, and accumulation of metabolic waste products may all be driving forces for the development of HAND. While further validation is needed, proteomics and metabolomics studies show the potential of identifying new biomarkers and cluster of biomarker expression in different neurocognitive disorders that could potentially give new evidence about this new promising approach.

### MicroRNA

MicroRNAs (miRNAs) have been abundantly demonstrated in the brain, where they regulate synaptic plasticity and brain development, implying that miRNA dysregulation may parallel neurocognitive dysfunction. First, a study on HIV subjects compared miRNA profiling on tissue from frontal cortex and CSF in PWH with or without HAND to healthy controls (Pacifici et al., 2013). In this article, the authors showed a different expression of 66 miRNA in CSF, of which 35 were also found in the frontal cortex. Specifically, they identified four miRNA with identical expression in tissue in CSF and one miRNA 20-fold higher expressed in CSF compared to brain tissue. Analysis of significantly altered miRNA showed their role in remodeling of cytoskeleton, cellular adhesion, expression of chemokine, neurogenesis, axonal guidance, notch signaling, synaptogenesis, and nerve impulses.

In a pilot study by Kadri et al. (2015), a plasma miRNA signature was associated with NCI in a cohort of PWH cared at LSU Health Sciences Center (LSUHSC) HIV Outpatient Clinic (HOP). As they did not consider alcohol consumption in this study, they later analyzed the miRNA profiling in the setting of alcohol use disorders in newly recruited PLWHA at the LSUHSC HOP clinic (Wyczechowska et al., 2017). They found that alcohol use disorders can represent a confounding factor for miRNA profile linked to HAND. Moreover, they confirmed their previous analysis using plasma samples from the CHARTER study and, also considering differences in the two, they validated a miRNA profile including 15 miRNA pairs that differentiate cognitively impaired PWH in both sites, LSUHSC and CHARTER.

Evidence from these studies, despite the many variables affecting circulating miRNA and thus encoding as potential confounders, could serve as a rationale for the development of novel tools for diagnosis and monitoring of HAND.

## Conclusions

In the modern ART era, considering the benefit in life expectancy of PWH, it is crucial to reassess the pathogenesis of HAND and the influence of age, ART, and comorbidities. Several studies have been performed to identify quantitative CSF/blood biomarkers for HAND; however, many gaps remain to be addressed: (i) most studies are cross-sectional, thus lacking to demonstrate a predictive role for the biomarkers associated with NCI; (ii) NCI classification is often analyzed as classical categories (ANI, MND, and HAD), thus losing the possibility to define markers associated with the evolution of individual cognitive performance; (iii) a minority of works evaluated the effect of optimized ART on possible CSF/blood biomarkers associated to different neurocognitive behavior, thus lacking information on the potential use of these markers to guide ART use and selection.

Use of an approach aimed to explore multiple biomarkers could help clarify the most critical gaps in current HIV research, and it is likely that omics approaches (transcriptomics, proteomics, and metabolics) will accelerate HAND biomarker discovery and validation with a possible impact on the development of adjunctive treatment and monitoring of CNS disease.

## Author Contributions

AB, LT and GB: conception and design of study, database creation, acquisition of data, drafting of article and critical revision of the final manuscript. AM, JR, TB and AG: drafting of article and critical revision of the manuscript. All authors gave final approval to the submitted manuscript.

## Conflict of Interest Statement

The authors declare that the research was conducted in the absence of any commercial or financial relationships that could be construed as a potential conflict of interest.
